# Transition Metal Complexes of Naproxen: Synthesis, Characterization, Forced Degradation Studies, and Analytical Method Verification

**DOI:** 10.1155/2016/3560695

**Published:** 2016-02-29

**Authors:** Md. Sharif Hasan, Ruhul Kayesh, Farida Begum, S. M. Abdur Rahman

**Affiliations:** ^1^Department of Clinical Pharmacy and Pharmacology, University of Dhaka, Dhaka 1000, Bangladesh; ^2^Department of Pharmaceutical Chemistry, University of Dhaka, Dhaka 1000, Bangladesh

## Abstract

The aim of our current research was to synthesize some transition metal complexes of Naproxen, determine their physical properties, and examine their relative stability under various conditions. Characterizations of these complexes were done by 1H-NMR, Differential Scanning Calorimetry (DSC), FT-IR, HPLC, and scanning electron microscope (SEM). Complexes were subjected to acidic, basic, and aqueous hydrolysis as well as oxidation, reduction, and thermal degradation. Also the reversed phase high-performance liquid chromatography (RP-HPLC) method of Naproxen outlined in USP was verified for the Naproxen-metal complexes, with respect to accuracy, precision, solution stability, robustness, and system suitability. The melting points of the complexes were higher than that of the parent drug molecule suggesting their thermal stability. In forced degradation study, complexes were found more stable than the Naproxen itself in all conditions: acidic, basic, oxidation, and reduction media. All the HPLC verification parameters were found within the acceptable value. Therefore, it can be concluded from the study that the metal complexes of Naproxen can be more stable drug entity and offer better efficacy and longer shelf life than the parent Naproxen.

## 1. Introduction

Compounds with metals as therapeutic agents for various diseases states have been investigated in the last few decades [1–3]. Metals can react with different atoms of many amino acids residues in proteins providing therapeutic actions [4]. Because of their different mechanism of actions, the development of metal complexes for various drugs provides an alternative route of novel drug delivery system [5]. Binding of a drug to metalloelement can enhance or reduce its activity and in some cases the complex may have even such activity that the parent compound does not have [6].

**Figure 1:**
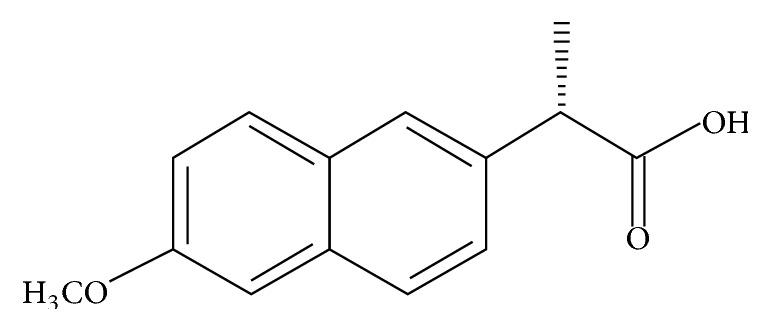
Chemical structure of Naproxen.

Nonsteroidal anti-inflammatory drugs (NSAIDs) are some of the most prescribed drugs worldwide as antipyretic, analgesic, and anti-inflammatory agents [7]. However, the major limitation to NSAID use is the gastric and intestinal mucosal damage [7]. In the UK an estimated 12000 peptic ulcer complications and 1200 deaths per year are attributable to NSAIDs use [8]. Therefore, much has been studied so far to reduce the gastric toxicity of NSAIDs and in this regard, complex formation of NSAIDs with transition metals has long been recognized as an effective way of reducing gastric mucosal lesions caused by these drugs [9]. Thus, the present study is performed to synthesize transition metal complexes of Naproxen (Figure 1), to resolve their characterization, and to observe their relative stability by conducting forced degradation studies. Forced degradation is an integral component of validating many analytical methods that indicate stability of the drug and detect different impurities coming from manufacturing processes [10, 11]. They facilitate analytical methodology development and validation, better understanding of stability of drug molecules in different environments, and finding out the degradation pathways of drugs and byproducts [12–14].

To the best of our knowledge, a combined study of synthesis, characterization, and forced degradation study of Naproxen-metal complexes has never been done yet. But completed studies of the degradation of the drug substance and drug product are required at the new drug application (NDA) stage. So in our current study we put our effort to synthesize and characterize different transition metal-Naproxen complexes along with the determination of their relative stability under various stressed conditions. Also, the RP-HPLC method for analysis of Naproxen outlined in USP has been verified for the drug-metal complexes.

## 2. Experimental

### 2.1. Materials

All the apparatus and reagents were in analytical grade of Merck origin, used without purification, and were available in the laboratory of the Department of Clinical Pharmacy and Pharmacology, Faculty of Pharmacy, University of Dhaka.

### 2.2. Synthesis of Sodium Salt of Naproxen (HL)

0.82 gm (0.1 M) of Naproxen (ligand) was dissolved with 0.1 M of sodium hydroxide solution in water to form the sodium salt of Naproxen. Then the solution was sonicated for 5 minutes and kept in room temperature. The potency of Naproxen must be considered before preparation. The reaction mixture was put on a water bath to evaporate until a crystal film appeared; upon cooling the white product separated out.

### 2.3. General Procedure for Synthesis of Transition Metal Complexes

Equimolar metal salts dissolved in water were added to the above mixture so that the ratio *n (metal) : n (ligand)* of monovalent, divalent, and trivalent ions used was 1 : 1, 1 : 2 and 1 : 3, respectively, in each case and immediate precipitation occurred. Then the solid complexes were isolated by filtration, washed until being free of chlorides with the corresponding solvent (methanol or water), and finally dried at room temperature.

### 2.4. Analytical Methods

HPLC system (Shimadzu Prominence), equipped with UV-visible detector, was used for the analysis of the samples. LC-solutions software was used for recording data. Reversed phase C-18 column (Zorbax Eclipse XBD-C18, 150 × 4.6 mm, 5 *μ*m) was used to analyze the standards and samples. The HPLC assay method described in United States Pharmacopoeia (USP35 NF30, volume 3, Page 3996, 2012) was used to analyze the samples.

### 2.5. Forced Degradation Conditions

It is important that more strenuous conditions than those used for accelerated studies (25°C/60% RH or 40°C/75% RH) should be used while performing this study. In general, the following conditions were investigated: (1) acid and base hydrolysis, (2) hydrolysis at various pH, (3) thermal degradation, (4) photolysis, and (5) oxidation. It was focused on determining the conditions that degrade the drug by approximately 10%. However, beginning at extreme conditions (80°C or even higher, 0.5 N NaOH, 0.5 N HCl, 3% H_2_O_2_) and testing at shorter (2 hours, 5 hours, 8 hours, 24 hours, etc.) multiple time points allow for a rough evaluation of degradation rate. The conditions listed in Table 1 were followed in the current study [15].

### 2.6. Acid Hydrolysis

1 mg/mL of solutions was prepared of each Naproxen-metal chelates. Then 1 mL of sample solutions and 4 mL of 1 M HCl were mixed and mixture was kept for 24 hours at room temperature. After 24 hours, the sample solutions were allowed to be neutralized by 1 M NaOH to pH 7.0 and the volume was made up to 10 mL with diluting solution. The prepared samples were then analyzed on HPLC.

### 2.7. Basic Hydrolysis

1 mg/mL of solutions was prepared of each Naproxen-metal chelates. Then 1 mL of sample solutions and 4 mL of 1 M NaOH were mixed and mixture was kept for 24 hours at room temperature. After 24 hours, the sample solutions were allowed neutralized by 1 M HCl to pH 7.0 and the volume was made up to 10 mL with diluting solution. The prepared samples were then analyzed on HPLC.

### 2.8. Oxidation

Oxidation of Naproxen and its metal complexes was studied using 10% H_2_O_2_ for 24 hours. 1 mL of samples and 9 mL of 10% H_2_O_2_ solution were mixed and the mixture was kept for 24 hours at room temperature. After 24 hours, the sample solutions were analyzed.

### 2.9. Reduction

Reduction of Naproxen and its metal complexes was studied using 10% Sodium bisulfite for 24 hours. 1 mL of samples and 9 mL of 10% Sodium bisulfite solution were mixed and the mixture was kept for 24 hours at room temperature. After 24 hours, the sample solutions were analyzed.

### 2.10. Water Hydrolysis

1 mL of samples and 9 mL of distilled water were mixed and the mixture was kept for 24 hours at room temperature. After 24 hours, the sample solutions were analyzed.

### 2.11. Dry Heat Degradation

5 mg of each Naproxen-metal chelate was placed in an oven for 3 hours at 105°C and then the heated samples were dissolved in 5 mL of diluting solution and allowed to attain the room temperature. The prepared samples were then suitably diluted and analyzed.

### 2.12. Analytical Method Verification

The analytical method was verified according to United States of Pharmacopeia (USP) 37, General Information, 1225, Validation of Compendial Procedures guideline with respect to some parameters used in method validation. According to 21 CFR 211.194(a)(2) of the current Good Manufacturing Practice regulations, “suitability of all compendia testing methods used shall be verified under actual conditions of use” (USP 37, General Information/*〈*1226*〉* Verification of Compendial Procedures). In current case, system suitability, solution stability, accuracy, precision, and robustness were performed for method verification.

## 3. Result and Discussion

### 3.1. Physical, Analytical, and Thermal Properties

**Figure 2:**
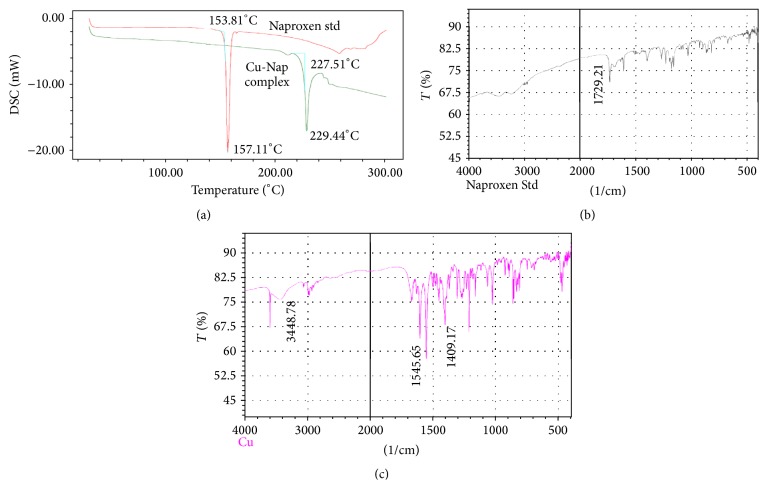
(a) DSC thermogram of Naproxen and its Copper complex: (b) and (c) IR spectrum of Naproxen and its Copper complex, respectively.

All the complexes synthesized were crystalline solids and soluble in common organic solvents but insoluble in ethanol and acetone. They were characterized by elemental analyses, IR spectra, thermal analysis, electronic photography (SEM), and magnetic properties (NMR).  Table 2 shows the results of elemental and thermal analysis of the complexes. The melting points or decomposition temperatures of the chelates are higher which suggests their thermal stability. Naproxen decomposes at 153°C where the complexes decompose in the range of 218–250°C (Figure 2(a)) followed by complete burning at above 700°C. The representative equations for the formation of the complexes can be presented as

(1) Mn+Cln·hH2O+nNaL=MLn·mH2O+nNaCl+h−mН2O

(where M = Co, Cu, Zn, Ag, Fe; *n* = 1 or 2 or 3; *h* = 0, 2, 4, or 6; *m* = 0 or 2 or 3).

### 3.2. FTIR Spectra

In this study, the carboxylic acid group of Naproxen shows the *ν*(C=O) stretching mode as a band at *ν* = 1729 cm^−1^. This was gone because of deprotonation and in the sodium salt there were two new bands at 1535–1546 and 1405–1414 range, the carboxylate antisymmetric and symmetric vibrations, respectively (Figures 2(b) and 2(c)). The coordination of the carboxylate ion to metal ion took place in three different ways [16]. The difference between *ν*_as_(COO) and *ν*_s_(COO) in monodentate complexes was expected to be greater than 350 cm^−1^. When 200 < Δ*ν* < 350 cm^−1^, anisobidentate was observed which means an intermediate state between monodentate and bidentate and when Δ*ν* < 200 cm^−1^, the carboxylate groups were regarded as bidentate [16]. These situations were observed in the relative position of the antisymmetric and symmetric stretching vibrations. The main IR bands in the spectra of the sodium salt and the complexes are listed in Table 3. There was a band observed in the region 3145–3455 which is certainly due to the absorption of crystal or coordination water. Assignment of the carboxylate group of these metal complexes coordination depended on the position of both *ν*_as_ and *ν*_*s*_ bands and the values of Δ*ν* [17–21]. The values of all of these complexes lie in the range of 132–159 cm^−1^ which is close to that of sodium salt of Naproxen indicating that the carboxylate group acts as a bridging ligand. In solid state, the synthesized complexes of carboxylate mostly form bridged dimers (M_2_L_2_ or M_2_L_4_) and also polymeric networks [22].

### 3.3. NMR Spectra

In the ^1^H-NMR spectrum of Naproxen, the protons of methyl (CH_3_) group have a sharp doublet at ~*δ* 1.5–1.6; the methenyl (–CH) proton has a triplet around *δ* 3.60–3.90. In case of the methoxy (CH_3_O) protons, they exhibit a sharp singlet at *δ* 4.00 and the naphthyl protons appear at *δ* 7.10–7.80 as a multiplet. Sequentially all of these protons shift upfield in complexes; the methenyl proton displays the highest shift *δ* 0.25–0.30, whereas the methoxy protons shift the least ~*δ* 0.05. This occurs because of the lesser electron withdrawing capacity of metal ions in the complexes relative to that of the carboxy proton in the ligand. The hydrogen atom of the –COOH group is absent in the metal complexes of ^1^H-NMR spectra (range of 10–13 ppm). This data indicates coordination and the carboxyl group is not protonated and the complexation reaction takes place.

### 3.4. Scanning Electron Microscopy

**Figure 3:**
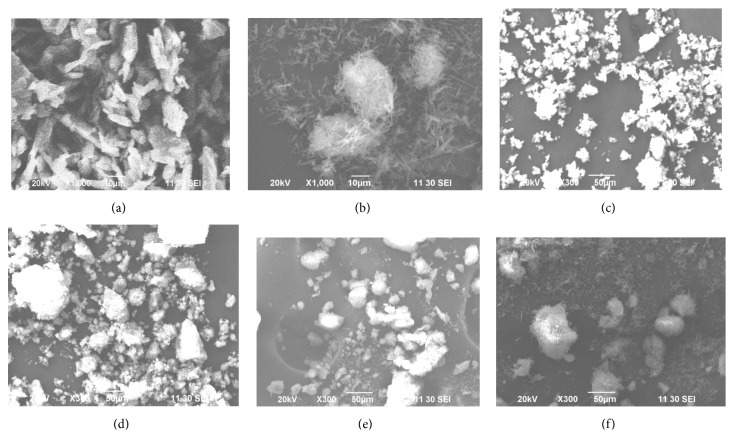
Scanning electron microscopy (SEM) photomicrograph of (a) Naproxen, (b) Naproxen-Copper complex, (c) Naproxen-Cobalt complex, (d) Naproxen-Iron complex, (e) Naproxen-Silver complex, and (f) Naproxen-Zinc complex, respectively.

Scanning electron microscope (SEM) images were taken in order to study the surface morphology of Naproxen-metal complexes. The SEM micrographs of ligand and its complexes are shown in Figure 3. The images showed particles with fiber-like morphology of the complexes compared to ligand (Naproxen) which is homogenously distributed in the solid powder. The photograph clearly indicated that the complexes are hydrated and they formed dimer or even polymeric networks in micrometer range.

### 3.5. Characterization by HPLC

**Figure 4:**
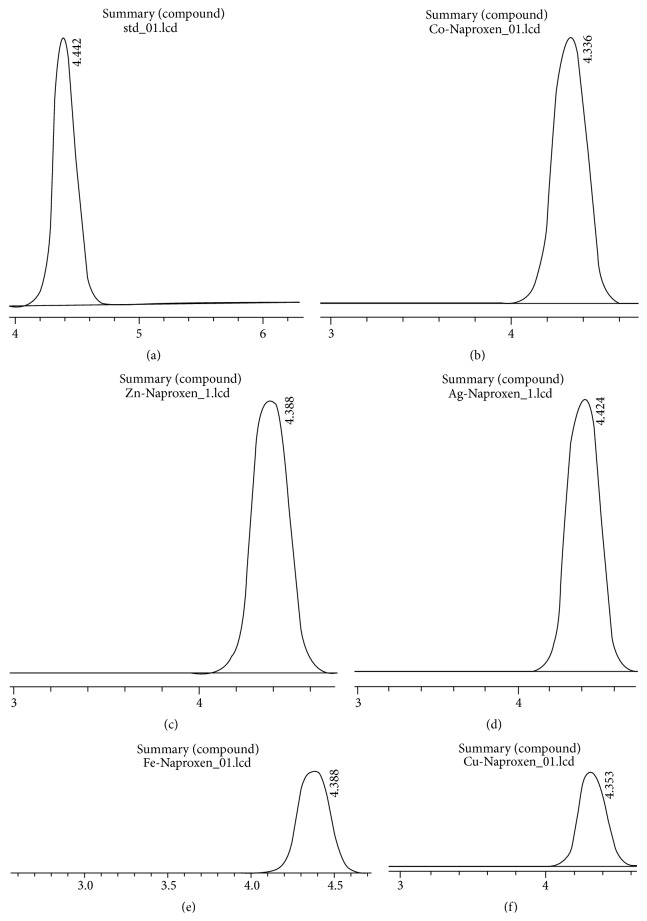
HPLC chromatograms of (a) Naproxen, (b) Naproxen-Cobalt complex (N-Co), (c) Naproxen-Zinc complex (N-Zn), (d) Naproxen-Silver complex (N-Ag), (e) Naproxen-Iron complex (N-Fe), and (f) Naproxen-Copper complex (N-Cu), respectively.

The RP-HPLC studies were performed in order to determine identity of the new synthetic products in comparison to the free ligand with respect to retention time. Acetonitrile and water in various ratios were used as mobile phases. HPLC methods were used to confirm the appearance of new products after the synthesis had been performed. The samples of ligand and complexes eluted close to each other with similar retention times (Figure 4). The chromatographic data for the complexes and free ligand are given in Table 4.

### 3.6. Stability Profile

**Figure 5:**
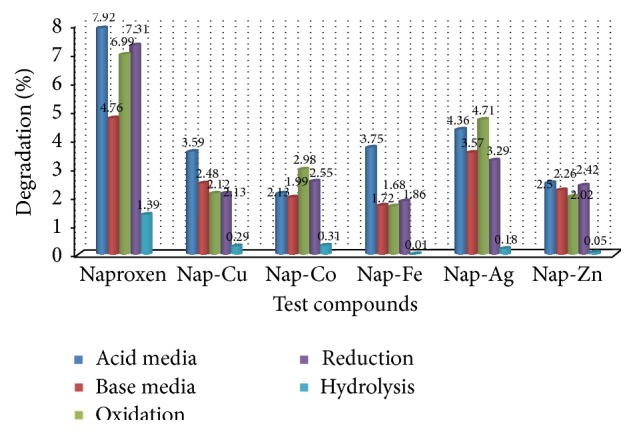
Graphical presentation of liquid state degradation of Naproxen-metal chelates.

**Figure 6:**
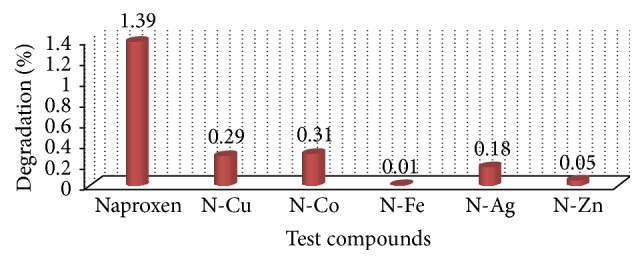
Graphical presentation of dry heat degradation of Naproxen-metal chelates.

In the forced degradation study it was found that Naproxen-metal complexes were the most stable compounds against any type of forced degradation condition applied than parent Naproxen. The highest degradation of Naproxen was found by acid hydrolysis and it was only 7.92%. Among the complexes, Naproxen-Iron complex was found most stable against the stressed conditions. Degradation levels are very close among all these complexes and it is due to the almost same coordination environment of the complexes. The most probable reason for their higher stability than Naproxen is the possibility of forming dimer or even polymer structures that is shown in SEM images. In DSC study, it was also revealed that the complexes have very high decomposition point than that of the parent Naproxen. That is why they are able to show better stability against stressed condition. The results were summarized in Table 5 and in Figures 5 and 6.

### 3.7. Method Verification Study

#### 3.7.1. System Suitability Test

All parameters were found within the limit. Results were summarized in Table 6.

#### 3.7.2. Solution Stability

Area changes were investigated up to three consecutive days. Low quantity of % RSD of area changes demonstrated that the drugs were fairly stable in the diluting solution and in the mobile phase. Results were shown in Table 7.

#### 3.7.3. Accuracy and Precision

Accuracy or recovery study was performed and result found in acceptable range for all samples for different concentrations. The range of the acceptability for accuracy was 97.0–103.0%.

The %RSD values found in precision study depicted in Table 8 showed that the compendial method provides acceptable intra- and interday variations for samples.

#### 3.7.4. Robustness

Predetermined variations were performed under the experimental conditions to assess their robustness. We changed pH ±0.2, flow rate ±50%, wave length ±3 nm, and solvent concentration ±30%. Results were shown in Table 9. No significant changes were found.

## 4. Conclusion

Search for drugs of higher efficacy and lower toxicity is a never ending effort. In our current research we were able to synthesize some Naproxen-metal derivatives and to highlight their stability profile under stressed conditions with a view to facilitating the invention of novel NSAIDs with better therapeutic efficacy. Lower toxicity of Naproxen in terms of gastric irritation has been established earlier. But from the result of present study further useful information was achieved that the metal derivatives of Naproxen were found more stable than Naproxen itself. This finding suggests that the metal derivatives of Naproxen can be more potent anti-inflammatory agent in human body with longer half-life as well as in the dosage form with longer shelf life when compared to the parent Naproxen.

## Figures and Tables

**Table 1:** Types of degradation reactions and conditions.

Degradation reaction	Typical conditions
Elevated temperature	Exposed to 105°C heat, up to 3 hours
Acid hydrolysis	Treated with 1 N HCl up to 24 hours
Base hydrolysis	Treated with 1 N NaOH up to 24 hours
Oxidation	Treated with 10% H_2_O_2_ solution up to 24 hours
Reduction	Treated with 10% Na bisulfite solution up to 24 hours
Water hydrolysis	Treated with water up to 24 hours

**Table 2:** Physical, analytical, and thermal properties of metal complexes.

Compound	Formula	Formula weight	Color	Yield % (g)	Analysis (%) calculated (found)			Melting point (°C)
					C	H	M	
(C_14_H_13_O_3_)_2_Cu·2H_2_O	C_28_H_30_O_8_Cu	558.07	Green	84.7	60.21 (60.08)	5.38 (5.32)	11.39 (11.42)	227.51
(C_14_H_13_O_3_)_2_Co·2H_2_O	C_28_H_30_O_8_Co	553.45	Light red	75.8	60.71 (60.39)	5.42 (5.45)	10.65 (10.67)	242.62
(C_14_H_13_O_3_)_3_Fe·3H_2_O	C_42_H_45_O_12_Fe	797.63	Yellow	86.3	63.19 (63.12)	5.64 (5.63)	7.00 (6.97)	235.13
(C_14_H_13_O_3_)Ag·H_2_O	C_14_H_15_O_4_Ag	355.13	White	85.3	47.30 (47.22)	4.26 (4.22)	30.38 (30.41)	218.89
(C_14_H_13_O_3_)_2_Zn·2H_2_O	C_28_H_30_O_8_Zn	559.9	White	78.3	60.01 (59.78)	5.36 (5.32)	11.68 (11.73)	225.28

**Table 3:** Infrared data (cm^−1^) of Naproxen-metal complexes in KBr.

Compound	*ν*_as_(COO^−^)			*ν*(H_2_O)
	*ν*_as_	*ν*_s_	Δ*ν*	
NaL	1545	1409	136	—
CuL_2_·2H_2_O	1554	1405	149	3406
CoL_2_·2H_2_O	1562	1415	147	3418
FeL_3_·3H_2_O	1601	1449	142	3422
AgL·H_2_O	1610	1451	159	3388
ZnL_2_·2H_2_O	1544	1412	132	3386

**Table 4:** Retention times of the complexes and their ligands.

Compound	Retention time (*t*_*r*_) (min)
Naproxen (ligand)	4.442
Naproxen-Copper complex (N-Cu)	4.353
Naproxen-Cobalt complex (N-Co)	4.336
Naproxen-Iron complex (N-Fe)	4.388
Naproxen-Silver complex (N-Ag)	4.424
Naproxen-Zinc complex (N-Zn)	4.388

**Table 5:** Degradation profile of Naproxen and its metal chelates.

Tested compounds	% of loss in acid media	% of loss in basic media	% of loss in oxidation	% of loss in reduction	% of loss in water hydrolysis	% of loss in dry heat
Naproxen	7.92	4.76	6.99	7.31	1.39	5.03
Nap-Cu complex	3.59	2.48	2.12	2.13	0.29	3.50
Nap-Co complex	2.13	1.99	2.98	2.55	0.31	3.07
Nap-Fe complex	3.75	1.72	1.68	1.86	0.01	2.65
Nap-Ag complex	4.36	3.57	4.71	3.29	0.18	4.18
Nap-Zn complex	2.50	2.26	2.02	2.42	0.05	3.78

**Table 6:** System suitability parameters.

Parameter	Naproxen (free ligand)	USP limit [23]
Tailing factor (*T*)	0.899	*T* ≤ 2
Theoretical plates (*N*)	2834.541	*N* ≥ 2000
% RSD of retention time	0.075	% RSD ≤ 2.0%
% RSD of peak area	0.094	% RSD ≤ 2.0%

**Table 7:** Solution stability parameters.

Days	Storage condition	Naproxen	Nap-Cu	Nap-Co	Nap-Fe	Nap-Ag	Nap-Zn
Freshly prepare sample	NA	611646	674528	648965	719744	639765	639976
Solution stability test (Day 2)	RT (% RSD of area change)	611199 (0.07%)	674141 (0.06%)	645675 (0.51%)	718188 (0.22%)	638212 (0.24%)	639233 (0.12%)
	Fridge (% RSD of area change)	611333 (0.05%)	674312 (0.03%)	648723 (0.04%)	719499 (0.03%)	639387 (0.06%)	639691 (0.04%)
Solution stability test (Day 3)	RT (% RSD of area change)	610387 (0.21%)	673102 (0.21%)	642671 (0.97%)	717297 (0.34%)	632037 (1.21%)	637441 (0.40%)
	Fridge (% RSD of area change)	611301 (0.06%)	674199 (0.05%)	648164 (0.12%)	719123 (0.09%)	638754 (0.16%)	639501 (0.07%)

RT: room temperature.

**Table 8:** Accuracy and precision parameters.

Parameters	Limit	Naproxen	Nap-Cu	Nap-Co	Nap-Fe	Nap-Ag	Nap-Zn
Accuracy	97.0–103.0%	98.95–100.11	99.34–99.87	98.45–99.24	98.45–101.45	99.45–101.37	99.55–102.62
Precision (intraday)	% RSD ≤ 2	0.812	0.522	0.657	0.729	0.478	0.835
	Day 1	0.974	0.745	1.135	1.256	0.875	0.435
Precision (interday)	Day 2	0.486	0.467	0.409	1.145	1.155	1.479
	Day 3	1.134	1.324	1.421	1.678	1.137	1.146
Different analyst and instruments	% RSD ≤ 3 (interday)	1.342	1.421	1.241	1.289	1.231	1.123

**Table 9:** Robustness of the method.

Parameter	Samples	Retention time (min)	Tailing factor	Theoretical plate
Flow rate (±50%) (*n* = 3)	Naproxen	3.121–6.675	0.564–1.134	2536–3002
	Nap-Cu	2.858–7.456	0.637–1.046	2535–3257
	Nap-Co	2.936–7.136	0.635–1.145	2504–2993
	Nap-Fe	3.012–6.546	0.537–1.267	2245–2866
	Nap-Ag	2.789–7.245	0.563–1.046	2536–3658
	Nap-Zn	2.977–6.896	0.682–1.035	2546–3527

Solvent ratio (±30%) (*n* = 3)	Naproxen	3.234–6.789	0.456–1.464	2454–3013
	Nap-Cu	3.567–5.346	0.523–1.368	2575–2935
	Nap-Co	3.786–5.359	0.684–1.257	2576–2896
	Nap-Fe	3.008–5.289	0.473–1.147	2689–3035
	Nap-Ag	3.678–6.149	0.568–1.427	2736–3024
	Nap-Zn	3.789–7.899	0.564–1.139	2524–3467

pH of buffer solution (±0.2) (*n* = 3)	Naproxen	3.934–4.567	0.836–0.956	2890–3205
	Nap-Cu	4.003–4.678	0.845–0.899	2600–3765
	Nap-Co	3.612–4.568	0.786–0.876	2535–3402
	Nap-Fe	3.613–4.257	0.823–0.956	2546–3867
	Nap-Ag	3.583–4.945	0.726–0.915	2394–3957
	Nap-Zn	3.993–4.456	0.736–0.925	2356–2895

Detector wave length (±3 nm) (*n* = 3)	Naproxen	4.346–4.467	0.823–0.913	2536–3406
	Nap-Cu	4.456–4.789	0.823–0.834	2675–3177
	Nap-Co	4.345–4.456	0.864–0.900	2465–2794
	Nap-Fe	4.412–4.467	0.789–0.822	2575–2904
	Nap-Ag	4.367–4.467	0.844–0.878	2356–3602
	Nap-Zn	4.367–4.419	0.812–0.901	2531–3387

Temperature (±10°C) (*n* = 3)	Naproxen	4.324–4.456	0.823–8.678	2567–2958
	Nap-Cu	4.326–4.567	0.746–8.134	2296–3042
	Nap-Co	4.324–4.467	0.783–0.899	2515–3657
	Nap-Fe	4.389–4.498	0.823–0.843	2549–3208
	Nap-Ag	4.358–4.418	0.823–0.845	2285–3647
	Nap-Zn	4.329–4.475	0.813–0.843	2576–3102
